# How China’s new health reform influences village doctors’ income structure: evidence from a qualitative study in six counties in China

**DOI:** 10.1186/s12960-015-0019-1

**Published:** 2015-05-05

**Authors:** Shengfa Zhang, Weijun Zhang, Huixuan Zhou, Huiwen Xu, Zhiyong Qu, Mengqi Guo, Fugang Wang, You Zhong, Linni Gu, Xiaoyun Liang, Zhihong Sa, Xiaohua Wang, Donghua Tian

**Affiliations:** School of Social Development and Public Policy, China Institute of Health, Beijing Normal University, 19 Xinjiekou Wai St., Beijing, 100875 China

## Abstract

**Background:**

In 2009, health-care reform was launched to achieve universal health coverage in China. A good understanding of how China’s health reforms are influencing village doctors’ income structure will assist authorities to adjust related polices and ensure that village doctors employment conditions enable them to remain motivated and productive. This study aimed to investigate the village doctors’ income structure and analyse how these health policies influenced it.

**Methods:**

Based on a review of the previous literature and qualitative study, village doctors’ income structure was depicted and analysed. A qualitative study was conducted in six counties of six provinces in China from August 2013 to January 2014. Forty-nine village doctors participated in in-depth interviews designed to document their income structure and its influencing factors. The themes and subthemes of key factors influencing village doctors’ income structure were analysed and determined by a thematic analysis approach and group discussion.

**Results:**

Several policies launched during China’s 2009 health-care reform had major impact on village doctors. The National Essential Medicines System cancelled drug mark-ups, removing their primary source of income. The government implemented a series of measures to compensate, including paying them to implement public health activities and provide services covered by social health insurance, but these have also changed the village doctors’ role. Moreover, integrated management of village doctors’ activities by township-level staff has reduced their independence, and different counties’ economic status and health reform processes have also led to inconsistencies in village doctors’ payment. These changes have dramatically reduced village doctors’ income and employment satisfaction.

**Conclusions:**

The health-care reform policies have had lasting impacts on village doctors’ income structure since the policies’ implementation in 2009. The village doctors have to rely on the salaries and subsidies from the government after the drug mark-up was cancelled. China’s national health reforms are attempting to draw village doctors into the national health workforce, but the policies have impacted their income and independence. Further research into these concerns and monitoring of measures to adequately compensate village doctors should be undertaken. Reasonable compensation strategies should be established, and sufficient subsidies should be allocated in a timely manner.

**Electronic supplementary material:**

The online version of this article (doi:10.1186/s12960-015-0019-1) contains supplementary material, which is available to authorized users.

## Background

Human health resources are crucial to building sustainable health systems worldwide [1]. The ability of a country to achieve universal health coverage depends largely on the motivation and deployment of health workers who are responsible for delivering health services [2]. Studies on the severe shortage and maldistribution of health workers have been conducted in recent years, especially in rural and remote areas [3,4], and the severe shortage of health workers is a critical issue that must be addressed through policy, planning and implementation of innovative strategies, especially in Pacific and Asian countries [5]. How to attract, retain and motivate health workers in rural areas is a great challenge in the world, especially in low- and middle-income countries, and the underlying reasons for the shortage of health workers should be explored. Previous studies have shown that payment systems influence the motivation and retention of the primary care providers [5-7]. In rural China, village doctors act as the backbone of the medical system, performing basic medical care and public health services [8-11]. A good understanding of village doctors’ income is essential for the government to develop appropriate strategies to provide strong incentives to improve health workers’ productivity and retain staff in rural areas and form the basis for performance assessment and performance-based financing of village doctors’ work [12,13].

**Table 1:** **The basic characteristics of sampling counties**

**Geographical location regions**	**County**	**Num of village doctors interviewed**	**GDP per capita (RMB)**	**The proportion of rural population (%)**	**Farmers’ per capita income (RMB)**	**Hospital beds (per 1,000 capita)**	**Doctors (per 1,000 capita)**	**Village doctors (per 1,000 rural residents)**
South-eastern China	Changshu in JiangSu Province	9	123,882^a^	61.67^a^	19,467^a^	4.55^b^	2.99^b^	1.16^b^
Middle China	Xichuan in HeNan Province	8	41,690^a^	79.08^a^	13,487^b^	1.09^b^	1.60^b^	1.21^c^
Middle China	Xinjian in JiangXi Province	10	44,925^a^	77.36^a^	9,018^a^	2.27^a^	1.29^b^	1.53^b^
North-eastern China	Acheng in HeiLongJiang Province	7	42,211^a^	59.10^a^	9,817^a^	2.51^b^	1.23^b^	1.37^c^
South-Western China	Pingchang in SiChuan Province	8	9,524^a^	80.49^a^	6,115^a^	1.60^a^	1.60^a^	1.94^b^
North-western China	Maiji in GanSu Province	7	19,586^a^	53.21^a^	3,825^a^	2.97^b^	1.35^c^	1.60^c^

Source: ^a^Data from the Statistical Communique of sampling counties on the 2012 National Economic and Social Development.

^b^Online data (in 2012) published by the health bureaus of sampling counties.

^c^Data (in 2012) provided by the health bureaus of sampling counties.

**Table 2:** **Demographic characteristics of village doctors interviewed (** ***N*** **= 49)**

**Variables**	***N*** ** (%)**
*Gender*	
Male	34 (69.4)
Female	15 (30.6)
*Age*	
≤40	8 (16.3)
41 ~ 50	15 (30.6)
51 ~ 60	16 (32.7)
>60	10 (20.4)
*Years of working as a village doctor (year)*	
≤20	10 (20.4)
21 ~ 40	29 (59.2)
>40	10 (20.4)
*Education*	
Junior college or above	6 (12.2)
Secondary school or below	43 (87.8)
*The manner of obtaining the highest degree of certification*	
Prior to employment	15 (30.6)
On-the-job training	34 (69.4)
*The way of practising medicine*	
Western medicine	27 (55.1)
The traditional Chinese medicine	4 (8.2)
Combined therapy of Chinese and western medicine	18 (36.7)
*Total*	*49 (100)*

**Table 3:** **Village doctor’s income structure among different counties**

**County**	***Salary***	***Allowance for basic public health services***	***Remuneration for drug sales***	***General fee for medical service***	***Profits from drug sales***	***Other incomes***
ChangShu	Y	Y	N	Y	N	N
XiChuan	N	Y	Y	Y	Y	Y
XinJian	N	Y	Y	Y	N	Y
Acheng	N	Y	Y	Y	Y	Y
PingChang	N	Y	Y	Y	N	Y
MaiJi	N	Y	N	N	Y	Y

In China, the barefoot doctor system was introduced in the mid-1960s, whereby health workers were recruited from among the farmers, given limited training and supported through the Cooperative Medical Scheme (CMS) to provide preventive and basic health services and referral to higher level units, often while still engaged in farming work [14]. Prior to implementation of this policy, in 1955, CMS was established to underwrite the cost of health care for the rural population through the communes [14]. By the end of the 1960s, a three-tiered system of health providers in rural areas has been successfully established [15]. These public health achievements made China an international model for primary health care for the World Health Organization’s Declaration of Alma-Ata [16]. At that time, services in rural area were basic, but population coverage for health services was high, and services were generally accessible. Barefoot doctors were selected and supported by their villages, and they were accountable to the village committee [17]. They earned their income by generating “work points (*gongfen in Chinese*)” with medical services, similar to farmers doing agricultural work, and their income was counted by converting time for medical service to similar time for agricultural work [8,15,18]. There were no incentives for individual innovation or for profiteering.

The economic reforms of the 1980s signalled a transition from the planned economy towards the Socialist-Market Economy, and in the rural areas, there was a major shift in decision making and financial responsibility for farming from the village (or commune) to the family [9,14]. With this shift, the financial base for the CMS disappeared. The original barefoot doctor system, called village doctors after 1985, collapsed in 1981 because they lost their political and financial support [19]. From that time on, village doctors started charging patients for their services [20]. Because of the new economic incentives, they began to shift their focus to treatment rather than preventative care [7]. Although village doctors could obtain subsidies for their work on preventive health, anti-epidemic work, maternal and child health and other administrative affairs, it was only a small part of their income, averaging 5% to 10% [21-23]. Health workers were forced to seek alternative sources of income for their survival, and a fee-for-service system became the dominant way to pay village doctors [17].

With the changes in epidemiological patterns, the government realized the importance of strengthening rural health systems and developing community-based primary care in China [24-28]. To improve the overall function of the rural health service network, the Minister of Health proposed the Integrated Management of Township Health Centers (THCs) and village clinics in 2002. Integrated Management was further developed in 2009, and THCs are in charge of the management of village clinics, including medicine, personnel, finance, facilities, routine work, etc. In the meantime, the New Rural Cooperative Medical Scheme (NRCMS) was developed and became widespread after 2003 [15,29]. Since then, the level of medical insurance for rural residents has continued to increase, and therefore, rural residents have become more likely to visit a doctor.

In 2009, the Chinese Central Government proposed the establishment of the National Essential Medicines System (NEMS) [29,30], which covers drug production, pricing, distribution, procurement, prescribing and payment and instigated a “zero-mark-up” policy for medicines on the new National Essential Drugs List when they were prescribed at the grassroots level [31]. Concurrently, the National Basic Public Health programme was launched for all residents, and the THCs and village clinics had to provide time-consuming basic public health services to rural residents. Health policy emphasized the shift in tasks from treatment to public health services to strengthen the rural health system and fight chronic disease. Over 5 years, changes had been made to village doctors’ work roles and their income structure, but these changes had not been clearly clarified and studied.

Health policies continue to impact village doctors’ income structure. A good understanding of how China’s health reforms since 2009 are influencing village doctors’ income structure is very important. It will assist authorities to adjust related polices and ensure that village doctors’ employment conditions enable them to remain motivated and productive. This study examines key policy-related influences on village doctors’ incomes, functions and satisfaction by focusing on the sources and changes in their income since these health reforms were introduced.

## Methods

### Qualitative study

This qualitative study was based on in-depth interviews with village doctors between August 2013 and January 2014. Interviews were conducted in Chinese by a team of six experienced researchers with training in the interview process.

#### Sampling

A stratified multi-stage cluster sampling method was used to select the sample [32]. During the first stage, Jiangsu Province (southeast), Henan Province (middle), Jiangxi Province (middle), Heilongjiang Province (northeast), Sichuan Province (southwest) and Gansu Province (northwest) were selected from the 34 provinces or municipalities of China. Second, one county or district was chosen from each province or municipality as follows: Changshu county from Jiangsu, Xichuan county from Henan, Xinjian county from Jiangxi, Acheng district from Heilongjiang, Pingchang county from Sichuan and Maiji district from Gansu. Third, three towns were selected from each county, taking into account heterogeneity in economic development. Fourth, three village clinics were selected from each town, and only the village clinic directors were interviewed. Although the geographic distribution and socioeconomic status were taken into account in the sampling process, the sampling steps were not random in this study. More detailed information about the sampling locations is shown in Table 1. Overall, 49 of 54 village doctors (village clinic directors) from 6 counties of 6 provinces participated in the survey, and the response rate was 90.7%. The demographic characteristics of the participants are shown in Table 2. All participants were provided with an information leaflet and consented to be interviewed. The study protocol was approved by the Ethical Review Board of School of Social Development and Public Policy at Beijing Normal University [32].

#### Data collection

All the interviews were conducted in village clinics using a pre-prepared, open-ended interview schedule. The interview schedule (showed in Additional file 1) was divided into the following two parts: basic information (age, gender, education, the qualification for medical practice, year started working as a village doctor, the manner of obtaining their highest degree of certification and the way village doctor practises medicine) and the open-ended questions (including administration and management, roles and workload, income and income structure, changes of income structure, policy factors influencing income structure and perspectives on the health-care policies). Six interviewers conducted the interviews, which took 30–60 min per village doctor. All the interviewers were trained for 36 h by the leader of the research group, and an instruction and coding manual was provided to each interviewer who took part in this study. All the interviews were audio recorded, and all the detailed information of the interviews was categorized by research assistants. After the interviews, the audio data were transcribed verbatim and filed by researchers and research assistants.

#### Data analysis

Thematic analysis was used to analyse the qualitative data between April 2014 and May 2014. All team members read the materials thoroughly several times to obtain a sense of the whole. Afterwards, key words and themes were identified and coded. The coding differences were resolved through thorough discussion to ensure that all perspectives on the themes were represented in the results. All team members took part in the discussions about the themes and subthemes of key policy factors influencing village doctors’ income.

## Results

### Village doctors’ income components

Previous studies showed that village doctors’ income structure varied in the different geographic locations [33,34]. In this study, village doctors’ incomes were divided into the following six components (Table 3):

1. *Salary*: Money that employees receive for doing their job, especially professional employees working in an office, usually paid every month, not including benefits, bonuses or any other potential compensation;
2. *Allowance for basic public health services*: To provide each rural resident with free National Basic Public Health Services (NBPHS), central and local governments have subsidized public health programmes since 2009, and the level of funding has risen year by year, from 15 RMB (US$ 2.4) in 2009–2010 to 25 RMB (US$ 4.0) in 2011–2012, to 30 RMB (US$ 4.8) in 2013, and to 35 RMB (US$ 5.6) per capita in 2014 [35], and village doctors can obtain approximately 40% of the funds for NBPHS, which means 6–14 RMB per capita in the corresponding year would be allocated to village doctors according to the current policy [35,36];
3. *Remuneration for drug sales*: According to NEMS, government-owned primary health-care providers must sell essential medicines with zero-mark-up [31], which means they are not allowed to charge the patients a mark-up, but village doctors are entitled to a government payment of about 15% of total drug sales to compensate for the loss of the drug mark-ups [36]. While remuneration for drug sales is generally calculated on the basis of total drug sales, there is always a ceiling on the total government payment;
4. *General fee for medical service*: In the village clinics that had implemented NEMS, registration fee, checkup fee, injection fee (including intravenous infusion fees, excluding drug costs) and dispensing fee were merged into the general medical fee for medical service. Commonly, the fee for medical services and prescriptions is about 5–10 RMB of which the patient pays 20%, and the rest is paid through the NRCMS. [35-37]. The patients should pay up the one fifth general fee for medical services in most areas and seek reimbursement for drugs. Also, there is always a ceiling in total from the government. If remuneration for drug sales exceeds the ceiling, there will be no further payment from the government;
5. *Profits from drug sales*: Quite a few village doctors still sell drugs with a mark-up secretly because of insufficient financial resources of the local government;
6. *Other income*: Allowance from local governments or collectives and non-medical income, such as money from farming and other jobs.

It is important to note that the implementation of the national policy in different sampling locations has not been simultaneous. Therefore, the practical situation differs significantly from county to county in China, and village doctors’ income structure also varied with sampling location in this study (Table 3). Furthermore, differences in income structure also occurred in the same place as a result of lax supervision of the local government.

### Factors that influence village doctors’ income structure

From in-depth interviews, the following four main factors influencing village doctors’ income structure were found: the integrated management of rural health organizations including THCs and village clinics, the NEMS, NBPHS and NRCMS. As a result of the comprehensive effects of these four policies, drug mark-up was cancelled gradually, and village doctors had to rely on salaries, remuneration for selling essential medicines, the allowance from the NBPHS and the general fee for medical service from the government.

#### Integrated management

The integrated management of rural health organizations was an essential factor determining village doctors’ income structure in China. In 2009, integrated management was restored, that is, THCs began to be responsible for the management of the village clinics again, including medicine, personnel, finance, facilities, routine work, etc. Specifically, all work related to the medical services of village doctors was under the supervision and management of THCs. In reality, the organization and implementation of integrated management had a decisive effect on village doctors’ income structure. However, the process of implementation of integrated management varies in different sampling counties.

As we found in Changshu county, which is the third of China’s 100 wealthiest counties in 2014 [38], with a GDP per capita of 123,882 RMB (US$ 19,953.6) in 2012, integrated management has been rigorously implemented. Village clinics, which are owned by the village collective, are regarded as the affiliated agencies of THCs, and the village doctors are strictly managed by THCs in many aspects, such as facilities, routine work, medicine and finance. Village doctors received a salary, an allowance for NBPHS, remuneration for selling essential medicines and a general fee for their medical service from NRCMS according to the payment systems of the THCs. In addition, village doctors’ working hours were regulated, and they were not allowed to perform agricultural or other side activities for other income. It is important to note that there was a fairly good collective economic foundation in Changshu county; the village clinics have never been completely free from the collective ownership of the village and have been under the supervision of their village committee. Understandably, the implementation of the integrated management of rural health organizations policy in 2009 was relatively easy and better compared with other counties in this study.

In contrast, in Maiji district, which is a state poverty county with per capita GDP of 19,586 RMB (US$ 3,154.7) in 2012, village doctors still functioned as self-employed workers. Only those who were selected as public health workers to perform public health services could obtain an allowance from the NBPHS and general fee for medical service from the government. There was minimal supervision of village doctors’ routine work, drugs and finance from THCs, and they did not receive a salary. Although the policy on NEMS was also implemented in Maiji district, there was no strict supervision. Village doctors could still make a profit (mark-up of 15%) from selling essential drugs on the National Essential Medicine List because the remuneration for selling essential medicines was not given to the village doctors in a timely manner. Occasionally, some village doctors received a small remuneration. As a village doctor said, “NEMS exists in name only here”.

In some counties, a performance appraisal was used to monitor and manage village doctors’ routine work. Specifications for NEMS and NBPHS were regarded as the basis for the payment of subsidies. If the village doctors could not meet the quality requirement, they would lose part of their subsidies*.* The performance appraisal was mainly conducted by THCs; in other words, THCs act as both the player and the referee during the process of performance appraisal. Thus, the assessment was often unfair because of the conflicts between village doctors and THCs.

*It is difficult to achieve these goals (set by superiors), so we can get only a part of the allowance. (in Xinjian county)*

*They are both the referee and players, how can we win then (to get full allowance fairly from township health workers). (in Acheng district)*

In the past, village doctors were practising personally. In integrated management, THCs are in charge of the management of village clinics, including medicine, personnel, finance, facilities and routine work. Village doctors act more similar to workers in THCs, which means they should comply with the provisions of village clinics and THCs. They could not leave their post in village clinics without permission for other activities as before, and their absenteeism would be trigger a warning or penalty. In some areas, village doctors needed to work full-time and were on duty in village clinics, so some revenue-generating activities,such as farming, were limited.

*We must be on duty, or we will be fined. (in Changshu county)*

*In the past, during busy farming seasons, I was always going to do farm work for my family, but now, I can’t. (in Xinjian county)*

#### National Essential Medicines System

The main purpose of the NEMS was to improve population access to essential medicines, reduce medical costs and guide doctors to use medicines safely, especially in primary rural medical institutions [31,39]. The NEMS oversees drug production, pricing, distribution, procurement, prescribing and payment [39]. According to NEMS, only drugs in the National Essential Medicine List (NEML) were permitted in THCs and accessible to village doctors and must to be sold with zero-mark-up, but doctors could obtain remuneration for drug sales (at approximately a 15% mark-up) for drug sales from the government [30,36,40]. However, the actual implementation is quite different among the six counties in this study. For example, we found that no non-essential drugs were used in the village clinics in Changshu county, while non-essential drugs were still used in many village clinics and sold with mark-up in village clinics in Maiji district and Acheng district.

Funds for remuneration for NEMS come from governments at all levels but were assigned at the county level in accordance with the regulations established by each county government. The amount of funds and allocation strategies differed largely among different counties due to the different economic situations and administration consciousness of the local government, especially that of the top leadership. For example, village doctors could obtain 3,000 RMB per month in Changshu county on average, while only a few hundred Yuan were allocated to village doctors in Maiji district. Compared with the past, when a village doctor could receive much more than the 15% drug mark-up, their medical income fell dramatically in most areas.

*Before, drug profits could be 30,000-40,000 RMB a year, but now there is less than 10,000 RMB from drug profits instead (in Xinjian county)*

*In the past, drug profits were our major revenue, about half of our income. Now, we can only get a limited amount of drug subsidies from the government. It is even not enough for us to make a living. (in Xinjian county)*

In the village clinics that had implemented NEMS, village doctors could get remuneration for drug sales. Moreover, the allocation of the remuneration differed greatly in different areas, due to varied historical, social and economic conditions. In some areas, there was also a subsidy ceiling based on the budget.

*I could get about 12,000 RMB last year according to the provision, but only 4,000 RMB was assigned to me. I have asked for it many times, but no response. (in Acheng* district*)*

After the implementation of the NEMS in 2009, only drugs on the NEML (307 species) were allowed to be used. In the village clinics, only about 60–120 were used. The full range of pharmaceuticals required by the NEML was not available in many areas. Drug supply was also difficult in many counties especially in mountain areas as a result of lack of profits. The species and total amount of essential medicines were not enough for daily treatment in village clinics, resulting in a decrease in patients at village clinics.

*We have no effective drugs, so a large proportion of patients went to higher level hospitals. (in Xinjian county)*

*Not enough drugs, we couldn’t treat them, and after a long time they won’t trust me anymore. (in Xinjian county)*

*There were no antifebrile/antipyretics, especially for children, we cannot even cure a cold. No patients, how can we make a living? (in Changshu county)*

There are exceptions of course. In some areas (Xichuan county and Pingchang county) where village doctors’ treatment was mainly based on the Traditional Chinese Medicines (TCMs), the implementation of NEMS had little impact on their revenues. Because TCMs were not limited by NEMS, village doctors could profits from drug sales by selling TCMs as before.

*The national essential drugs system has no influence on my income, (because) I only use a few western medicines. (in Pingchang county)*

*Most village doctors, here, can treat diseases with TCMs, so the effects of the national essential drugs system on our income were not obvious. I can treat about 30 patients every day, and my total annual income was about 60,000 RMB. Of course, I was not the highest-paid village doctor. (in Xichuan county)*

#### National Basic Public Health Services

Promotion of the equalization of basic public health services had been China’s focus in health-care reform since 2009. It aimed to ensure that urban and rural residents had equal access to basic and the most effective public health services, to narrow the gap between urban and rural residents in basic public health services and further to ensure that urban and rural residents get sick less often. Meanwhile, *The National Public Health Service Standard* was published, and it provided a framework to assess the capacity and performance of public health systems and public health governing bodies. Furthermore, the implementation of NBPHS was monitored and managed as an obligation and mandatory task by local governments.

*I have to do these public health services, because superiors will check on our working progress. If not completed, we will lose part of bonus or even be punished. (in Xinjian county)*

The Chinese government had also committed to increasing government funding for NBPHS, so the government subsidy per person increased from 15 RMB in 2009 to 30 RMB in 2013 [37]. Village doctors could obtain approximately 40% of the allowance for basic public health services after completing the relevant work. The subsidy was managed at the county level in accordance with the principles of the local government. Primarily, the subsidy was released to THCs, which were in charge of the allocation of funds between themselves and the village doctors.

*In theory, we should get 12 RMB (40% of 30 RMB) per capita, but in fact, we only got about 8 RMB on average. (in Xinjian county)*

*I did not know the standard for issue of allowance, I got only 1300 RMB last year, but I was responsible for at least 2,000 residents. (in Xichuan county)*

Allowances for basic public health services were allocated to village clinics by population health services. Because the number of village doctors in each village clinic is different, the allowance for basic public health services distributed to each village doctor also varies.

*In our village, there is a total of 2,700 residents. If there are two village doctors, the allowance for basic public health services is not too small, but there are 4 village doctors (in our village), thus each of us could get only 800 RMB. …It is too little for us for living. (in Pingchang county)*

Village doctors had to perform basic public health services divided into 11 categories that covered 43 items in 2013 as specified by the government. At this stage, NBPHS mainly include the establishment of health records, health education, immunization, prevention and treatment of infectious diseases, chronic diseases (hypertension and diabetes) management, serious mental disease management, child health care, maternal health-care services, elderly health care, etc. A village doctor might provide basic public health services for 2,000 rural peasants at most, although there was often only one village doctor. There is no doubt that NBPHS increased their workload and reduced the time allowed to provide medical services.

*It (public health service) takes a large part of my work time, and less time is allocated to treatment. With less out-patient services, my medical revenue decreased a lot. (in Xinjian county)*

*To get more time for out-patient services, I have to do the public health services in the evening. Sometimes, that makes me exhausted. (in Xinjian county)*

#### New Rural Cooperative Medical Scheme

The Chinese government launched NRCMS in 2003. Participation in the NRCMS was voluntary, although farmers were actively encouraged to join. At its inception, the NRCMS aimed to provide health coverage for the nation’s entire rural population by 2010 [41]. Population coverage had extended rapidly; by the end of 2012, the NRCMS had been introduced in 2,566 counties, covered 98.26% of the rural population and has 805 million members [42]. In addition, the NRCMS was operated at the county level. The county health bureau or bureau of human resources and social security was responsible for the design, implementation, management and administration of the scheme. The origin of NRCMS revenues is threefold: they come from central and local governments and households. NRCMS revenues were earmarked for paying for health services, including part of the salaries and the operation costs of service providers. Investment using NRCMS funds was not allowed. The benefit package was determined by the funds available from government subsidies and household contributions. NRCMS funds were raised to a new level (no less than 290 RMB per capita in 2012), and part of NRCMS funds could be used for remuneration for drug sales according to the service provision. The total amount and payment methods of NRCMS had major impacts on where patients go for medical services. For example, in Xichuan county, the NRCMS funds were only 290 RMB in 2013, while it was 650 RMB in Changshu county. In Xichuan county, there was a reimbursement ceiling (50 RMB per capita) for outpatient service in village clinics, but not at THCs. Therefore, patients would go to THCs for a minor illness rather than to village clinics. For village doctors, fewer patients resulted in lower medical income.

*Patients would rather to go to township health centers for outpatient service, because they can get more reimbursement. (in Xinjian county)*

*In township health centers, at most 90 percent of the cost of out-patient treatment (most of which is drug expense) could be reimbursed, but only 50RMB at most could be reimbursed in our clinics. Naturally they choose township health centers, because their reimbursement was over 50 percent. (in Xichuan county)*

According to the policy, village doctors could receive a general fee for medical service from NRCMS funds for outpatient service. The fee was a fixed proportion of total NRCMS funds, and different counties developed specific reimbursement standards and distribution modes. For example, a village doctor could receive 5 RMB per visit or per prescription in Xinjian and Xichuan county, respectively, whereas village doctors could receive 10 RMB in Changshu county. To avoid excessive costs, a reimbursement ceiling was established, and the village clinics were monitored in areas such as outpatients, pharmaceutical consumption and the intensity and rationality of prescriptions.

*Excessive prescriptions were prohibited, and I could only get the limited general fee. (in Changshu county)*

*In the past, about 20 patients a day were treated in our clinic, but now, less than 10 outpatients were treated. …At the end of the month, we submit a list of prescriptions. After the prescriptions are checked out, we can get the general fees for medical service next month. (in Xinjian county)*

## Discussion

A previous study indicated that motivation is influenced not only by specific incentive schemes targeted at workers but also by the whole range of health sector reforms [43]. To better achieve the goals of health-care reform and to strengthen the rural health-care system in China, effects of policies on village doctors’ financial incentives should be assessed [44-46]. How to use innovative payment methods to serve the goals of health sector reform was an important issue [47]. In this study, we found that ongoing health system reforms had impacted village doctors’ independence and gradually transformed village doctors’ income structure. The integrated management of village doctors’ activities by township-level staff has reduced their independence and changed their roles. NEMS cancelled drug mark-ups, removing their primary income source. The government implemented a series of measures to compensate, including paying them to implement public health activities and provide services covered by social health insurance.

Health-care reform policies were set at national levels in a political process and were then communicated to subordinate levels which were then charged with managerial and administrative tasks of putting policy into practice. County government was the main body to perform the health-care reform policies; however, policy implementation was quite different among the six counties as a result of different management styles and socioeconomic status. Inconsistency of policy implementation had impacts on the village doctor’s behaviours and motivation. Poor governance and monitoring of village doctors’ behaviour and differing behaviour of THCs was another factor influencing the efficiency of policy implementation. As a synthesized effect, the ongoing health-care reform policies had gradually transformed village doctors’ income structure.

Accountability was a salient theme during the process of health-care reform [48]. As a main mechanism of accountability, integrated management played an important role in creating policy frameworks and was combined with effective oversight and regulation. Historically, as a special group, village doctors had always been self-employed peasants who lacked supervision [9]. When they lost institutional and financial support after the economic reform in 1978, many coping strategies were introduced such as over-prescribing drugs and injections, inflating drug mark-ups, informal user fees and ability to leave the workplace and perform agriculture work for income [5,49]. Under the integrated management system, the various coping strategies used in response to inadequate remuneration and poor supervision in the past were limited or prohibited [5].

The workers in THCs received regular medical education and were recruited by the government, they have *bianzhi* and the certificates of permanent urban residence (*Chengzhen Hukou*) and enjoy urban welfare. However, village doctors are farmers; they have the certificates of permanent rural residence (*Nongcun Hukou*) and could not become a regular employee of THCs. In fact, village doctors are temporary workers and are supervised by THCs. Their incomes were partly restricted, and their working time was also regulated. Thus, their income components and structure were also influenced. Although village doctors worked and were managed just similar to an outreach worker of THCs in some areas, it was hard for them to obtain the same benefits as a regular employee in THCs. In China, the Household Registration System (*Hukou*) prohibited the free migration of farmers to access the urban welfare system [45]. Strictly speaking, village doctor was not a formal professional title and could not be considered as a real doctor. The lack of legitimacy granted to village doctors by degreed health professionals was another barrier to defining their work roles and wage rate as workers in THCs [7,45]. The main objectives of the NEMS, to improve access to essential medicines and reduce the out-of-pocket expenditures of patients, had been achieved in some areas [31]. Village doctors’ prescribing practices changed following the introduction of the NEMS as a result of the short NEML [50]. All drugs on the NEML were restricted by the zero-mark-up policy, so the village doctor’s profits from drug sales were cancelled gradually. Both the zero-mark-up policy and the low availability of medicines could reduce the numbers of outpatients and their medical income to a certain extent [7,31,51]. Although there was an allowance for basic public health services, remuneration for drug sales and general fee for medical service, the extent to which these made up for the loss of income was unclear because there was lack of transparency and comparability of data on profits from drug sales. As profits from drug sales were cancelled, village doctors increasingly had to rely on salaries and subsidies from the government [40]. However, the local governments determined the companion policy and allocated budgets based on actual medicine costs, and the subsidy represented only the lost profit mark-up (15%). Incentives for over-prescription still exist [52] because a mark-up of at least 15% and sometimes far above 15% had been allowed previously.

As the superior authority, THCs also undertook part of the responsibility for NBPHS, about 60%, and could obtain the corresponding subsidy, so there is a conflict of interest between THCs and village doctors. In addition, the remuneration for drug sales and general fee for medical service for each village doctor were also distributed by THCs based on their assessment of the village doctor’s performance in providing public health services and medical services. This situation might cause misallocation and insufficient allocation to village doctors because some THC directors believed that village doctors have not performed their public responsibilities [11]. Of course, inadequate supervision of village doctors’ medical and public health services was also a reason for insufficient allocation in some areas.

Public health services are freely provided to residents, and all public health service items in rural areas were provided mainly by village doctors and THCs according to the national guidelines and performance standard for NBPHS. As more and more NBPHS items were imposed, although the government had increased financial input year by year, no new health workers were introduced to village clinics [50]. Village doctors therefore had to take more and more responsibilities and work under the NBPHS. Therefore, it decreased village doctors’ enthusiasm and reduced their time for profitable medical services. Village doctors noted that their work was not equally valued by the government but also showed their willingness to prioritize public health services if they received a more substantial subsidy [11]. Although the public health budget was tied to the annual performance assessment to further encourage village doctors to improve the quality of public health services, whether it can compensate for their loss of profitable activities and meet village doctors’ expectations remains unclear.

The NRCMS achieved great success in reducing out-of-pocket fee for medical services in the last few years [53], but whether it increased the number of outpatients in village clinics was difficult to determine [54]. Because of varied compensation strategies between village clinics and THCs, patients might be more inclined to go to THCs for economic reasons. In all sampled counties, village doctors could receive a general fee for medical service, but there was no unified standard to allocate this subsidy among the six counties.

It should be noted that compensation for NEMS and NBPHS was not being assigned in a timely and complete manner in some counties. The misappropriation and interception of funds was still widespread, especially in county- or city-level financial departments. A previous study [55] showed that China’s social sectors are heavily decentralized; the Ministry of Health has limited influence on the detailed design and implementation of health system reform at the sub-national level. The leaders of the local government in China were mostly driven by economic progress and revenue generation. However, health care was generally regarded as consuming, not generating, revenue. We also found that, if the head of the local government realized the importance of health reform, health policies were better implemented. Of course, in economically under-developed areas, it was a great burden for local governments to ensure the execution of these policies. Implementation of the policies had to be postponed because they were unable to provide enough resources, although the central government provided a great deal of resources. Therefore, the economic condition of the county or district also influenced the policy process.

To improve the provision of health-care services that the government wants, a number of alternative methods (such as a capitation payment, work-volume-based method and weight-based method) had been suggested for allocating government subsidies [7,56]. We suggested that, based on different contexts, reasonable compensation strategies should be established and monitored, and sufficient subsidies should be allocated in a timely manner [57].

The current study had a number of limitations. First, a total of 49 village doctors participated in the in-depth interview, and we were unable to represent the breadth and depth of each of their views here. Second, the sample was not random and may in fact bias towards a better situation than in poorer counties where the role of the village doctors may be more important. Third, although we aimed to presenting which and how health-care reform policies influence village doctors’ income structure, the extent of the effect of these policies on their income and the interactions among policies was not analysed because we believe that more quantitative research might be better to clarify these points, but we had not provided quantitative information—a major missed opportunity. We hope to perform a more quantitative study to further research village doctors’ financial incentives and how health-care policies influence them.

## Conclusions

The health-care reform policies have had lasting impacts on village doctors’ income structure since the policies’ implementation in 2009. The village doctors have to rely on the salaries and subsidies from the government after the drug mark-up was cancelled. China’s national health reforms are attempting to draw village doctors into the national health workforce, but the policies have impacted their income and independence. Further research into these concerns and monitoring of measures to adequately compensate village doctors should be undertaken. Reasonable compensation strategies should be established, and appropriate subsidies should be allocated in a timely manner.

## Additional file

Additional file 1:
**Interview template.** The template was divided into the following two parts: basic information and open-ended questions.
