# Supplementary material for: How China’s new health reform influences village doctors’ income structure: evidence from a qualitative study in six counties in China
Source: Hum Resour Health. 2015 May 5;13:26. doi: 10.1186/s12960-015-0019-1 (PMC4440293; doi:10.1186/s12960-015-0019-1)
Supplement: Additional file 1: — Interview template. The template was divided into the following two parts: basic information and open-ended questions. [file 12960_2015_19_MOESM1_ESM.doc]

**Interview template**

| **Name of Interviewer:** |  | **Date of Interview:** |  |
| --- | --- | --- | --- |
| **Interview Start & End Times:** |  | **Location:** |  |

**Demographic characteristics of village doctors**

| **Name:** |  | **Gender:** |  |
| --- | --- | --- | --- |
| **Birthdate:** |  | **Education:** |  |
| **Vocational qualification:** |  | **Years of working as a village doctor：** |  |
| **The manner of obtaining the highest degree of certification:** |  | **The way village doctor practice medicine** |  |

**Open Questions**

**Administration and management**

1. Could you describe how your clinics were administered and managed?
2. What are the changes in the administration and management compared with that before 2009?

**Roles and workload**

1. Could you describe your current job role in the rural health system?
2. Could you describe your job role before 2009?
3. What tasks do you perform now?
4. What tasks did you perform before 2009?
5. How do you feel about your workload compared with that before 2009?

**Income and income structure**

1. Could you tell us how much of your income last year?
2. Could you tell us how much of your income before 2009?
3. What does your income consist of last year?
4. What did your income consist of before 2009?

**Changes of income structure**

1. Is there any change in your total income compared with that before 2009?
2. Which part of income changed and how?
3. What are factors that lead to the changes of your income structure?

**Policy factors influencing income structure**

1. How do you feel about the health care reform in 2009? What opportunities or constraints influenced your income?
2. Which policy in the health reform influenced your income? How these policies influenced your income?

**Perspectives on the healthcare policies**

1. What are your expectations about your income?
2. What are your expectations about the healthcare policies?
